# Understanding the mechanisms of viral and bacterial coinfections in bovine respiratory disease: a comprehensive literature review of experimental evidence

**DOI:** 10.1186/s13567-022-01086-1

**Published:** 2022-09-06

**Authors:** Maria Gaudino, Brandy Nagamine, Mariette F. Ducatez, Gilles Meyer

**Affiliations:** IHAP, Université de Toulouse, INRAE, ENVT, Toulouse, France

**Keywords:** Bovine respiratory disease, respiratory viruses, respiratory bacteria, coinfections, cattle, bacterial superinfection, in vitro, experimental infections, influenza D virus

## Abstract

Bovine respiratory disease (BRD) is one of the most important diseases impacting the global cattle industry, resulting in significant economic loss. Commonly referred to as shipping fever, BRD is especially concerning for young calves during transport when they are most susceptible to developing disease. Despite years of extensive study, managing BRD remains challenging as its aetiology involves complex interactions between pathogens, environmental and host factors. While at the beginning of the twentieth century, scientists believed that BRD was only caused by bacterial infections (“bovine pasteurellosis”), we now know that viruses play a key role in BRD induction. Mixtures of pathogenic bacteria and viruses are frequently isolated from respiratory secretions of animals with respiratory illness. The increased diagnostic screening data has changed our understanding of pathogens contributing to BRD development. In this review, we aim to comprehensively examine experimental evidence from all existing studies performed to understand coinfections between respiratory pathogens in cattle. Despite the fact that pneumonia has not always been successfully reproduced by in vivo calf modelling, several studies attempted to investigate the clinical significance of interactions between different pathogens. The most studied model of pneumonia induction has been reproduced by a primary viral infection followed by a secondary bacterial superinfection, with strong evidence suggesting this could potentially be one of the most common scenarios during BRD onset. Different in vitro studies indicated that viral priming may increase bacterial adherence and colonization of the respiratory tract, suggesting a possible mechanism underpinning bronchopneumonia onset in cattle. In addition, a few in vivo studies on viral coinfections and bacterial coinfections demonstrated that a primary viral infection could also increase the pathogenicity of a secondary viral infection and, similarly, dual infections with two bacterial pathogens could increase the severity of BRD lesions. Therefore, different scenarios of pathogen dynamics could be hypothesized for BRD onset which are not limited to a primary viral infection followed by a secondary bacterial superinfection.

**Supplementary Information:**

The online version contains supplementary material available at 10.1186/s13567-022-01086-1.

## Bovine respiratory disease: the prelude of a respiratory outbreak

Bovine respiratory disease (BRD) is a general term for a range of respiratory disorders that can affect the lower respiratory tract in cattle. BRD is the second most common disease impacting the global beef industry, after neonatal calf diarrhoea [1], being a particular burden in young cattle and pre-weaned calves. Economic loss due to treatment costs, reduced performance (i.e. loss of weight or absence of weight gain, lighter carcass at slaughter or reduced milk production in dairy farms) and animal death can be substantial for producers [2]. Moreover, the high consumption of antibiotics to treat BRD causes concern over the emergence of antimicrobial resistance in cattle and also in humans, indirectly via the food chain, water, air, and manured and sludge-fertilized soils [3], thus threatening both animal and human health.

Early BRD manifestations include general signs, such as lack of appetite, self-isolation, depression and fever. These signs can evolve to more severe respiratory signs including nasal and eye discharge, salivation, rapid breathing, dyspnoea and prominent coughing [4]. BRD is known to be a multifactorial syndrome, triggered by a combination of environmental factors and infectious agents. Among environmental factors, events such as transportation and handling (i.e. for dehorning) are the most important stressful experiences for animals, as well as weaning or changes of feed [5, 6]. Cattle transportation alone is an important trigger in BRD, causing an increase in mortality during respiratory outbreaks, especially when following secondary bacterial infection [7]. Other environmental factors include the combination of insufficient ventilation, wet and dirty bedding, dust exposure and overcrowding, which can increase the possibility of pathogens transmission [8]. Also, the general microbial pressure in the environment due to lack of good hygiene practice can increase the risk of infections. Elements such as good colostrum quality and management, normal level of essential nutrients and adequate rest (especially after shipping) are essential for calves to maintain a normal immune function in response to challenging pathogens [9], as well as minimum stress exposure (i.e. good care when handling and using low stress techniques). Biosecurity measures (i.e. isolating new or sick animals and avoiding housing animals of mixed ages together) can also significantly decrease the risk of pneumonia outbreaks in cattle herds [9]. Lastly, routine feedlot vaccination can reduce the likelihood of primary viral infection, significantly reducing mortality [10]. In this review we will focus on the principal infectious agents involved in BRD and how the interactions between these pathogens impact pathogenesis.

## Most common infectious agents involved in BRD: from the twentieth century up to now

**Figure 1:**
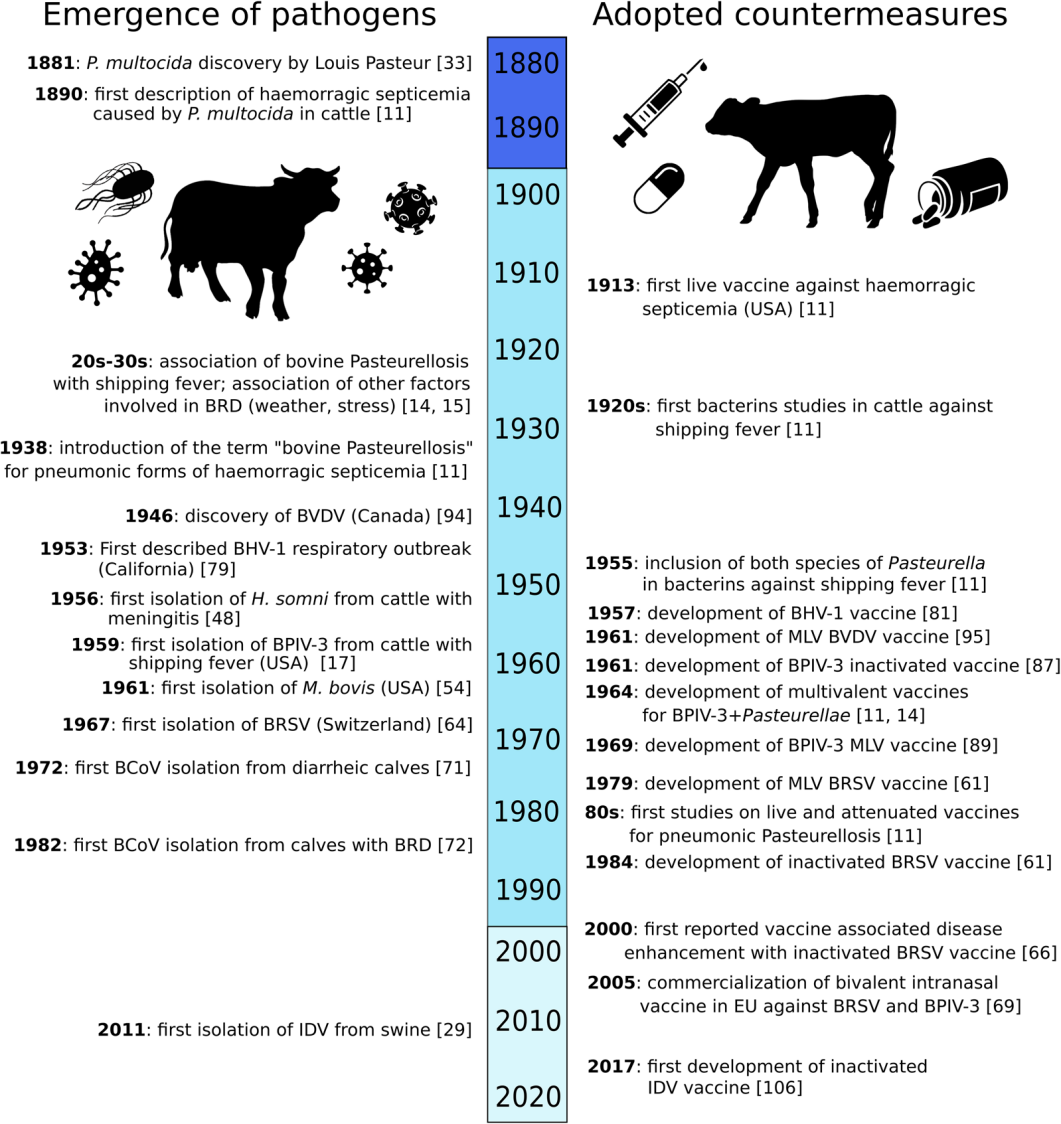
**Timeline showing examples on the history of BRD pathogens discovery and adopted countermeasures throughout the years.** An emphasis on vaccine countermeasures taken in Europe was given.

**Figure 2:**
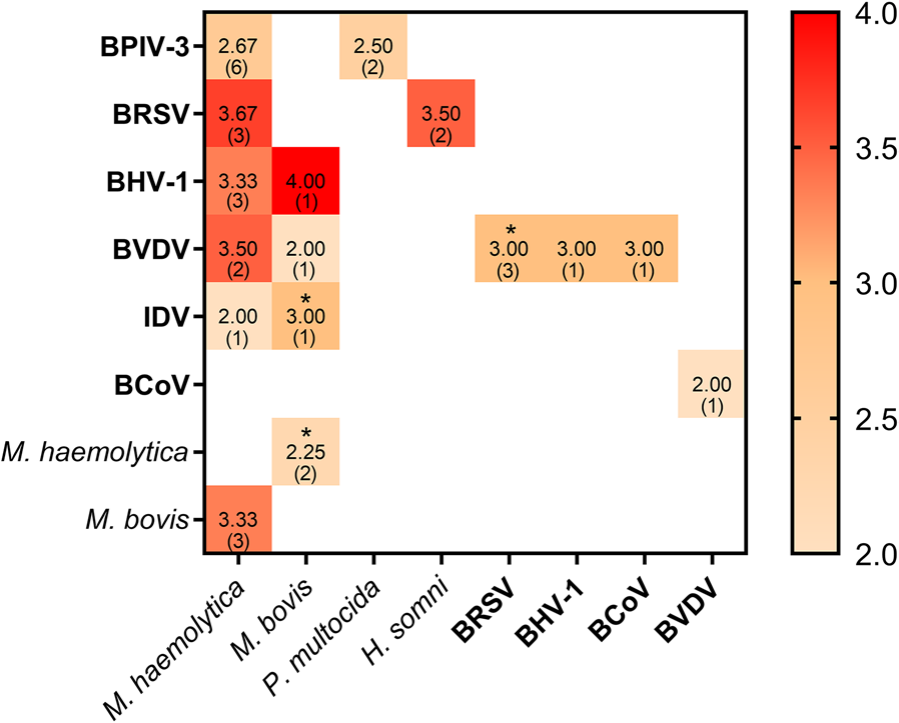
**Heat map showing the impact of sequential coinfections on respiratory pathology in cattle on in vivo experiments.** On the y-axis, the virus used for the primary viral infection is represented. On the x-axis, the pathogen used for the secondary superinfection is listed. The severity of coinfections on in vivo studies (compared to single pathogens) was given a score from 1 to 4 (colour code for the score is given in function of the increase in clinical signs, light orange to dark orange). The description of the scoring system that we used to describe the impact of coinfection in vivo is available as Additional file 1. Cell values represent the mean between the scores given to different in vivo studies performed with the same pathogens. The value in parentheses represents the number of trials carried out for each couple of pathogens that were used to calculate the mean score. White cells indicate an absence of in vivo studies for that specific couple of pathogens. *: the two pathogens were simultaneously inoculated in some studies.

At the beginning of the twentieth century, BRD was believed to be solely caused by bacterial infections and thus referred to as “bovine pasteurellosis” or, as reported in the first descriptions of the disease in late nineteenth century, as “haemorrhagic septicaemia” [11]. Around the 30 s’, scientists started to observe that beside *Pasteurella spp.* infection, other factors played a role in the disease development [12]. Animals experimentally inoculated with bacteria alone failed to reproduce the typical pneumonia signs [11, 13]. In addition, these bacteria could be cultured from apparently healthy animals after they were stressed such as during shipping (for this reason BRD was often referred as “shipping fever” during the last century) but also overcrowding, weaning and weather variations [14, 15]. In the 50 s’, the theory of viral causation gained support in North America, when bovine herpesvirus-1 (BoHV-1), the etiological agent of infectious bovine rhinotracheitis (IBR) [16], and bovine parainfluenza virus type 3 (BPIV-3), known as myxovirus parainfluenza 3 at that time, were isolated from cattle with shipping fever [12, 17]. During experimental infection, BPIV-3 mimicked natural pneumonia [18] with bacterial superinfections often accentuating the clinical signs and lesions in animals (Figures 1, 2).

BRD is now globally recognized as a polymicrobic disease, with bacterial coinfections known to affect the morbidity and mortality during viral respiratory infections [19]. Although the majority of pneumonia outbreaks are predominantly caused by bacteria and viruses, some fungi belonging to *Aspergillus* spp. genus [20] and parasites, commonly known as “lungworms” [21], can also trigger respiratory disease. Bacteria are generally isolated at higher prevalence in cattle with respiratory signs and because of this, antibiotic treatment is often the first choice made by veterinarian practitioners to avoid a rapid progression to severe BRD [22]. The most common bacteria isolated from cattle with respiratory signs belong to the *Pasteurellaceae* family, the most prevalent being *Pasteurella multocida*, *Mannhemia haemolytica* and *Histophilus somni* [23]. These three pathogens are also commensals of the upper respiratory tract (nasopharynx and tonsils) in healthy calves but can subsequently become opportunistic when host defences are compromised, leading to colonization of the lower respiratory tract [24]. Another class of bacteria that plays an important role in BRD belongs to the *Mycoplasmataceae* family, specifically the *Mycoplasma* spp. genus. Among these, *Mycoplasma bovis* is one of the most widespread, leading to the highest morbidity [25]. *Mycoplasma dispar* and *Mycoplasma bovirhinis* can be isolated from sick cattle as well [26, 27]. On the other hand, viruses also play an important role in BRD. Some viruses have been well known BRD agents for years and their pathogenesis is well characterized, whereas others have less clear roles. This list of viruses includes bovine respiratory syncytial virus (BRSV), bovine coronavirus (BCoV), bovine herpesvirus 1 (BoHV-1), BPIV-3 and bovine viral diarrhea virus (BVDV) [28]. Also, thanks to the advent of new generation of sequencing technologies (next generation sequencing (NGS)) new viruses have been discovered and could now be part of the official list of BRD pathogens, i.e. influenza D virus (IDV) [29–31]. Some viruses are thought to be more benign with an incidental finding during coinfection, but others such as BRSV can have a major pathogenic potential and can be the only etiological agent responsible for a respiratory outbreak in cattle herds, especially during the winter season [32].

To better understand the dynamic interactions between the various cattle respiratory pathogens, we will discuss the most common BRD-associated pathogens in the following paragraphs. Treatment options and preventive measures (i.e. vaccines) will also be covered for each pathogen.

### *Pasteurella multocida*

*Pasteurella multocida* is a Gram-negative bacterium that can infect a wide range of mammals and domestic birds. It was first discovered by Louis Pasteur around 1881 during the investigation of the etiological agent of fowl cholera [33]. Since the same bacteria could produce disease in different animal species, in 1939, scientists proposed to classify all these bacterial strains under the same genus and species, thereafter named *Pasteurella multocida* [34]. It is currently classified into five capsular groups (named from A to E) and 16 somatic serotypes (1 to 16). In cattle, *P. multocida* A:3 is the most common serotype isolated from animals displaying BRD and its pathogenicity has been confirmed in experimental studies [35]. In addition, serogroups B, E and F can be pathogenic in this species [36]. *P. multocida* infection in cattle can cause different types of bronchopneumonia, ranging from subacute to chronic fibrinopurulent but also fibrinous and fibro-necrotizing, which can be accompanied by a variable amount of intra-alveolar haemorrhage with moderate to severe neutrophils and macrophages infiltration in bronchi and bronchioles [37]. Vaccines to prevent *P. multocida* infection consist of bacterins (killed bacteria) [38] and the only available treatments are antibiotics, despite rising antibiotic resistance, as recently reported [39].

### *Mannheimia haemolytica*

*M. haemolytica* is another important Gram-negative bacterium involved in calf pneumonia. It was previously known as “*Pasteurella haemolytica*” but a revisitation of the *Pasteurellaceae* classification based on genetic similarity suggested its removal from the *Pasteurella* genus and thus the creation of a new genus named *Mannheimia* [40]. Hence, in this review, some scientific studies from before 1999 still contain the ancient nomenclature “*Pasteurella haemolytica*”. Currently, *M. haemolytica* is classified based on 12 capsular serotypes (named A1, A2, A5, A6, A7, A8, A9, A12, A13, A14, A16 and A17) [41]. Serotypes associated with respiratory disease in cattle are prevalently A1 and A6 [42]. Infected animals can first display general clinical signs such as fever along with loss of appetite and weight loss but also respiratory signs such as cough, nasal discharge and respiratory distress. The principal cause of death is acute fibrinous pleuropneumonia due to the obstruction of bronchioles and alveoli with fibrinous exudate [43]. Necropsy commonly reveals fibrinosuppurative pneumonia, necrotizing inflammatory response and alveolar damage and necrosis due to neutrophil and macrophage infiltration in the lung and fibrin deposition in the alveoli [41]. Vaccines containing *M. haemolytica* leucotoxin, its main virulent factor [44], are currently available. However, there is still a lack of data in the scientific literature to reinforce the full efficacy of this preventive measure [45]. Intranasal probiotic administration of *Lactobacillus* strain in order to prevent *M. haemolytica* colonization of the upper respiratory tract has been evaluated in a clinical trial and could represent a future possibility for the prevention of cattle pneumonia [46].

### *Histophilus somni*

*H. somni* is a Gram-negative bacterium that mainly affects cattle but can occasionally also infect small ruminants [47]. Unlike *P. multocida* and *M. haemolytica*, the circulating strains of *H. somni* are not currently classified into specific serotypes and no comprehensive nomenclature is available to date. It was first isolated in 1956 from cattle with meningitis [48]. Animals of all ages can be affected but recently, it was shown that weaned calves seem to be at higher risk of infection [49]. Although *H. somni* is considered, like the other mentioned *Pasteurellaceae*, a commensal bacterium of the nasal tract, different strains have also been isolated from urogenital secretions, which can be responsible for venereal spread [50]. When the bacterium colonizes lungs and gains access to the blood stream, it can cause systemic disease that is not limited to the respiratory tract. *H. somni* infection can thus also cause encephalitis, myocarditis and sudden death due to acute septicaemia [51]. Post-mortem findings in the lungs include bronchopneumonia and fibrinous pleurisy [52]. Diagnosis based on gross lesions is accompanied by bacterial culture and molecular testing. Treatment options include large-spectrum antibiotics such as florfenicol but, similarly to *M. haemolytica*, bacterins are currently available as preventive measure, although they have failed to demonstrate effective protection in vaccinated animals [53].

### *Mycoplasma bovis*

*M. bovis* is a particular type of bacteria that greatly differs from those we previously described. Its represents one of the most challenging bacterial BRD pathogens. First isolated in 1961 [54], *M. bovis* causes pneumonia outbreaks in calves and young cattle but also mastitis in dairy cows, as well as otitis and abortion [55]. Like all the other members of the *Mycoplasmataceae* family, it is the smallest known bacteria. It lacks a cell wall, making it naturally resistant to several classes of antibiotics [56]. Clinical signs of infected animals can include fever, depression, nasal discharge, shallow breathing and cough. Post-mortem findings include bronchopneumonia with characteristic caseous necrotic lesions and also fibrinosuppurative bronchopneumonia [57]. Once introduced to a farm (i.e. through contaminated animals), eradication is difficult due to its strong environmental resistance [58] and widespread herd dissemination through direct contact [57]. Being a persistent intracellular bacterium lacking a cell wall reduces the choice for antibiotic treatment, representing another obstacle for its elimination. In addition, other major challenges include high antigenic variability of surface glycoproteins and the ability to evade host immune system [59]. Treatment efficacy is questionable with treated animals relapsing after a few weeks, in part due to increased antibiotic resistance over time [60]. A few vaccines are currently commercialized in North America, consisting of bacterins which offer limited protection [59].

### Bovine respiratory syncytial virus

BRSV (also known as bovine orthopneumovirus) is one of the most important viral pathogens involved in BRD. It is a single-stranded RNA virus belonging to the *Pneumoviridae* family (order *Mononegavirales*) [61]. Although it is similar to the human respiratory syncytial virus (around 40% of nucleotide identity) [62], BRSV has only been diagnosed in cattle as well as wild and domesticated small ruminants [63] and it is not considered a zoonotic pathogen. The first report of BRSV infection in cattle dates from 1967 in Geneva, Switzerland [64], after which it spread to other countries. There are currently ten circulating lineages [65], as based on genotyping of a small immunogenic region in the glycoprotein G which is important for antibody recognition. The biological significance of the antigenic variation in this region might thus be relevant for vaccine efficacy [32]. BRSV has the highest pathogenic potential among all circulating viruses in cattle with clinical signs ranging from mild-moderate to subclinical. Less frequently, BRSV infection can progress to respiratory acute distress syndrome including fever, depression, decreased food intake, and dyspnoea with open-mouth breathing that can exacerbate during late stage infection [66]. In some cases, up to 80% of morbidity is reported, with mortality reaching up to 20% [67]. Emphysematous and haemorrhagic lung lesions, as well as necrotizing bronchiolitis and interstitial pneumonia, especially in the cranial lobes, are characteristics of BRSV infection at necropsy [66]. The infection can also produce the typical multi-nucleated syncytial cells formed by the fusion of several cells caused by the fusion protein F. Several vaccines are available on the market as a prophylactic measure against BRSV infection [68, 69].

### Bovine coronavirus

BCoV is a single-stranded RNA virus belonging to the *Coronaviridae* family (*Coronavirinae* subfamily, order *Nidovirales*), and is classified within the Betacoronavirus 1 subgroup (Embecovirus) [70]. It was first isolated in 1972 from diarrheic calves [71] and in 1982 from BRD calves [72]. Endemic in cattle worldwide, it is known for its pneumo-enteric tropism, causing both enteric disease (especially calf diarrhoea) and pneumonia outbreaks [73]. After experimental BCoV inoculation, colostrum-deprived calves develop cough, nasal discharge, respiratory distress and diarrhoea [74]. Treatment for the enteric disease associated with BCoV infection is largely limited to supportive care (i.e. rehydration, electrolyte administration, and the use of nonsteroidal anti-inflammatory drugs [75, 76]). Several vaccines against the enteric form are currently available [77]. Vaccines protecting against BCoV respiratory-associated disease are still missing.

### Bovine herpesvirus type 1

BoHV-1 is a DNA virus belonging to the *Herpesviridae* family (subfamily *Alphaherpesvirinae*, order *Herpesvirales*) and the known etiological agent for infectious bovine rhinotracheitis (IBR) [78]. It is believed to have been first isolated from German cattle with venereal disease in the nineteenth century and later associated with respiratory disease during a 1954 outbreak in California [79–81]. BoHV is divided into two circulating subtypes, BoHV-1.1 and BoHV-1.2 [82], which are both characterized by acute inflammation of the upper respiratory tract but can also sporadically cause abortion in cattle, as well as conjunctivitis, vaginitis and enteritis [83]. In particular, respiratory signs associated with BoHV-1 infection include mucopurulent nasal discharge (sometimes accompanied by ulcers in mouth and nose), conjunctivitis, coughing, sneezing, and difficult breathing [84]. BoHV-1 in cattle is characterized by lifelong latent infection with sporadic viral reactivation and shedding when immune defences are compromised (i.e. following a stressful event such as shipping) [85]. Commercially available vaccines are broadly used in various European countries to prevent BoHV-1 associated syndrome leading to progressive eradication of the disease as part of a monitoring program for control maintenance and eradication [86].

### Bovine parainfluenza type 3

BPIV-3 is a single-stranded RNA virus belonging to the *Paramyxoviridae* family (genus *Respirovirus*, order *Mononegavirales*) [87]. It was first isolated in 1959 from cattle with shipping fever and named “myxovirus shipping fever 4” (SF-4) [17, 88, 89]. BPIV-3 is now endemic, with three circulating genetic groups worldwide, named A, B, and C [90]. Infection with BPIV-3 usually leads to mild respiratory signs, such as fever, dry cough, nasal and ocular discharge, increased respiratory rate and dyspnoea [91, 92]. Infection of the upper respiratory tract can also lead to a transient immunosuppression, creating an opportunity for secondary bacterial superinfections [87], a component of calf enzootic pneumonia. Several vaccines are commercially available, often in association with BRSV [68].

### Bovine viral diarrhea virus

BVDV is a single-stranded RNA virus belonging to the *Flaviviridae* family [93]. It is a member of the genus *Pestivirus*, first discovered in North America during the 40 s’ and later isolated in 1957 [94, 95]. Two different Pestivirus species are currently in circulation, Pestivirus A (formerly known as BVDV-1) and Pestivirus B (formerly known as BVDV-2) [93]. Infection with BVDV often manifests as respiratory and gastrointestinal disease, the latter being associated with diarrhoea and mucosal disease (when a cytopathic strain is involved), especially during persistent infections [96, 97]. BVDV induces lesions of mucosal (especially intestinal) and lymphoid tissues that can result in acute diarrhoea, thrombocytopenia and respiratory signs [98, 99]. Its main role during BRD is immunosuppressive, paving the way for subsequent superinfections by other viral or bacterial respiratory pathogens. Vaccine prophylaxis via maternally derived antibodies has been shown effective at protecting cows and newborn calves but efforts are still to be made to eradicate the disease [100].

### Influenza D virus

Influenza D virus (IDV) is a single-stranded RNA virus belonging to the *Orthomyxoviridae* family (genus *Deltainfluenzavirus*, order *Articulavirales*). Like Influenza C (ICV), it has a segmented genome consisting of seven genomic segments, unlike Influenza A and B viruses (IAV and IBV) that harbour eight segments [29]. IDV was discovered in 2011, making it the most novel bovine respiratory pathogens to date [29]. Unlike the other genera of the *Orthomyxoviridae* family, IDV is most prevalently found in cattle, which is considered its primary host [101]. To a lesser extent, IDV can also infect small ruminants, swine and feral swine, camelids, horses and hedgehogs [102]. Several lines of evidence suggest that IDV can be zoonotic but to what extent is currently being investigated [101].

Different circulating IDV genotypes have been characterized through sequence analysis of the hemagglutinin esterase-fusion (HEF) segment, the most prevalent being “D/OK” and “D/660” with divergent lineages present in Japan, Canada and the United States of America [8, 103, 104]. IDV also seems to undergo genetic reassortments among its different lineages which is a common feature of influenza viruses [8]. Pathogenic differences amongst the different circulating strains remain questionable as IDV can be isolated from both sick and healthy animals and is often found alongside other pathogens in cattle with BRD signs [8]. Calves experimentally infected with IDV display mild to moderate signs of repeated spontaneous coughing, abdominal dyspnoea with increased respiratory rates, and abnormal lung sounds [105]. Upon necropsy, the lung tissue reveals subacute bronchointerstitial pneumonia with neutrophils in bronchial lumens, neutrophilic and macrophagic alveolitis, as well as microscopic alveolar lesions [105]. A vaccine that confers partial protection in cattle was developed in a research study but has not been commercialized [106].

### Other influenza viruses

The role of other influenza viruses in BRD still remains unclear to date. Natural infections of IAV virus in cattle have been reported, as well as few studies showing low seroprevalence of IAV infection in this species [107]. In addition, experimental challenges showed that cattle can develop moderate to severe clinical signs and seroconversion following IAV infection [107]. Despite all these pieces of evidence, cattle is not considered a host for IAV, unlike swine and avian species. Several reports described ICV detection in samples from sick cattle [108–110], suggesting its circulation in cattle population, similarly to IDV. However, studies of experimental infections in cattle are currently missing in literature and convincing proof of its pathogenicity and role in BRD in cattle are still to be provided.

## Prevalence of coinfections in cattle herds: an interplay between viruses and bacteria

RT-qPCR commercial kits and decreased NGS costs have made the detection of multiple respiratory pathogens from clinical samples simpler and cost effective. Today, BRD is recognized as a polymicrobial disease with numerous studies acknowledging the high frequency of coinfections. 50.73% of nasal swabs taken over a four-year period from Canadian cattle (*n* = 883) showing respiratory signs were positive for at least two respiratory pathogens [8], supporting a 2018 study, that detected at least two pathogens in 41% of the nasal swabs (*n* = 23) collected from steers during a respiratory outbreak in Brazil [111]. Bronchoalveolar lavages collected in Denmark from 46 healthy calves and 46 sick calves tested for respiratory pathogens revealed similar coinfecting pathogenic abundance. However, *H. somni* was the only pathogen that was positively associated to cattle with BRD [112]. In another study, lungs from Irish cattle with BRD were submitted for post-mortem examinations and dual infections were detected in 58% of lungs, with a high prevalence especially for *M. haemolytica* and *H. somni* coinfection [49]. The authors reported that *P. multocida* was the pathogen identified alone with the greatest frequency and the most frequently detected virus/bacteria coinfections were *P. multocida*/BPIV-3, *H. somni*/ BPIV-3, or *H. somni*/BRSV. Studies using metagenomics approaches on respiratory samples also confirmed that presence of multiple pathogens is more associated with illness than mono-infections. In a first study, the virome found in nasal swabs of 50 young dairy cattle with BRD was compared to 50 location-matched healthy control animals [30]. Viruses were detected in 68% and 16% of sick animals and healthy control animals, respectively. In addition, 38% of sick animals (versus 8% of controls) were infected with multiple respiratory viruses. Similar results were reported in another case–control study [110]. However, in another study that used a similar metagenomic approach, the authors failed in finding differences in terms of viral presence between sick and healthy animals in nasal swabs from feedlot cattle [31].

## Impact of coinfections on respiratory pathology in cattle: what is the experimental evidence?

### Viral and bacterial coinfections: the importance of primary viral infections preluding secondary bacterial superinfection

The occurrence of a primary viral infection followed by a secondary bacterial superinfection is the most common and well documented coinfection model of respiratory syndrome complex applied to cattle, swine [113], and humans [114]. Over the past 60 years, several studies have investigated the clinical ramifications of different bacterial and viral pathogenic interactions. The majority of the studies describes in vivo challenges during which young calves were inoculated with a viral pathogen followed by a bacterial superinfection a few days later. Most of the bacterial strains used belonged to the *Pasteurellaceae* family (*M. haemolytica*, *P. multocida* or *H. somni*), the classical etiological agents causing pneumonia in cattle. In two studies, *M. bovis* was concomitantly or subsequently inoculated after a viral strain. In this section, we comprehensively review the underlying mechanisms leading to enhanced pathogenicity during mixed respiratory infections in cattle. Table 1 summarizes the in vivo studies that were performed in calves to study the viral/bacterial respiratory coinfections. The description of the scoring system used to describe the impact of coinfection in vivo is available as Additional file 1.

**Table 1:** **In vivo studies from the scientific literature performed on young calves to assess the impact of virus/bacteria coinfection on BRD**

Reference	Primary viral challenge (route of infection and dose/animal)	Time between exposure to the two pathogens	Secondary bacterial challenge (route of infection and dose/animal)	Main results of the clinical trials	Impact of the coinfection on BRD (score 1 to 4)	Study limitations
Collier et al. [115]	BoHV-1	3 days	*M. haemolytica*	Coinfected group: longer duration of illness	3	
Hamdy et al. [15]	BPIV-3	6 h	*P. multocida*/*M. haemolytica*	BPIV-3 group: no respiratory disease, transient leukopenia Coinfected group: severe respiratory disease and pneumonic lesions	3	The animals were stressed, which could be a confounding factor
Saunders et al. [92] Trial 7	BPIV-3 (Intratracheal, 5 mL of 10^6^ TCID_50_/mL)	30 days	*P. multocida*/*M. haemolytica* (Intratracheal, 10 mL of 10^9^ CFU/mL)	BPIV-3 group: slight febrile response and leukopenia, nasal discharge and cough Exposure to *M. haemolytica* and *P. multocida* one month later did not provoke illness	2	Small number of animals (2), lack of mono-infected controls
Saunders et al. [92] Trial 8	BPIV-3 (Intratracheal, 5 mL of 10^6^ TCID_50_/mL)	Simultaneous	*P. multocida*/*M. haemolytica* (Intratracheal, 10 mL of 10^9^ CFU/mL)	BPIV-3 group: nasal discharge until day 3 Coinfected group: 40 °C fever and increased nasal discharge until day 11	3	Small number of animals (2), lack of non-infected controls
Baldwin et al. [116]	BPIV-3		*M. haemolytica*	Coinfected group: more severe respiratory symptoms upon subsequent exposure to *M. haemolytica*	3	
Collier et al. [117]	BoHV-1 (Intratracheal)	30 days	*M. haemolytica* (Aerosol)	Coinfected group: bronchopneumonia leading to the death of one calf	4	
Jericho et al. [118]	BoHV-1 (Aerosol, 10^6^ to 10^10^ TCID_50_/mL)	3 to 4 days	*M. haemolytica* (Aerosol, 5.5 × 10^5^–1.8 × 10^10^ CFU/mL)	Coinfected group: signs of bronchopneumonia ~ 4 days after virus exposure *M. haemolytica* group: no clinical signs	3	Viral and bacterial shedding were determined only after exposure; unclear number of animal/group
*Al-Darraji et al. [120, 124, 165, 166]	BRSV (Transtracheal, 20 mL of 2.9 × 10^4^ PFU/mL)	3 and 5 days	*M. haemolytica* biotype A serotype 1 (Transtracheal, 5 mL of 3 × 10^7^ CFU/mL)	*M. haemolytica* group: reduced physical activity BRSV group: inactivity, fever Coinfected group: inactivity, dry and intermittent cough, fever, increased respiratory rate, dyspnoea, anorexia, signs more pronounced in 5 day delayed group, loss of condition until the end of the experiment	4	
Yates et al. [119]	BoHV-1 (Aerosol, 10^7^ PFU)	4, 10, 20 and 30 days	*M. haemolytica* biotype A serotype 1 (Aerosol, 10^6^ PFU)	Coinfected group: higher fever, lung and pharyngeal lesions more severe in animals with a 4-day delay	3	Lack of control groups (non-infected, mono-infected)
Carrière et al. [120]	BPIV-3 (Aerosol, 100 mL of 5 × 10^4^ TCID_50_)	4 and 7 days	*M. haemolytica* biotype A serotype 1 (Aerosol, 100 mL of 10^12^ CFU)	All groups (BPIV-3, *M. haemolytica* and coinfected): no difference in terms of lung lesions, increase in rectal temperature and respiratory rate	2	
*Trigo et al. [124]	BRSV (Aerosol)	0, 3 and 6 days	*M. haemolytica* (Intranasal)	Virus or bacteria alone groups: mild clinical response BRSV + *M. haemolytica* superinfected group: increased pulmonary lesions; mono-infected groups: no observed lesions Coinfected group: higher rectal temperature compared to *M. haemolytica* group	3	
Potgieter et al. [121]	BVDV (Endobronchial inoculation)	5 days	*M. haemolytica* (Endobronnchial inoculation)	BVDV group: fever, nasal discharge, cough *M. haemolytica* group: mild signs Coinfected group: severe fibrinopurulent bronchopneumonia and pleuritis	3	
Potgieter et al. [129]	BRSV (Endobronchial inoculation, 10^8^ TCID_50_)	8 days	*H. somni* (Endobronchial inoculation, 10^7^-10^9^CFU)	BRSV group: no signs *H. somni* group: mild signs (fever, occasional cough and depression) Coinfected group 10^9^ CFU: severe clinical signs (mortality, diffused pneumonic lesions); 10^7^ CFU: mild clinical signs, less extended lung lesions	4	
*Sharma et al. [125]	BRSV (Intranasal)	6 days	*M. haemolytica* biotype A serotype 1 (Intranasal and intratracheal, 5 mL of 9 × 10^7^ CFU/mL)	Coinfected group: increased disease score, higher fever and higher mortality than both mono-infected groups	4	BRSV dose is not reported
Gånheim et al. [122]	BVDV (Intranasal, 2 mL of 10^6^ TCID_50_/mL)	5 days	*M. haemolytica* (Intranasal, 10 mL of 5 × 10^7^ CFU/mL)	Mono-infected groups: a few calves had fever and depression Coinfected group: all animals had fever and mild to severe depression, one calf did not recover, slower bacterial clearance, duration of elevated APPs lasted longer in coinfected group than BVDV but similar in *Mannheimia* group	4	
Gershwin et al. [128]	BRSV (Aerosol, 5 mL of 10^6^ TCID_50_)	6 days	*H. somni* (Intratracheal, (10 mL of 10^8^ CFU)	BRSV group: no lung lesions *H. somni* group: limited lung lesions Coinfected group: higher magnitude and duration of clinical signs, isolation of *H. somni* in lungs, extended lung lesions	3	Pathogen replication profile in animals is missing
Prysliak et al. [126]	BVDV (Intranasal aerosol^7^, 4 mL of 10 PFU/mL)	4 days	*M. bovis* (Intratracheal, 4 mL of 1.5 × 10^10^ CFU)	BVDV group: no lung lesions nor clinical signs, rectal temperature spike at 8 dpi Coinfected group: no lung lesions nor clinical signs	2	
Prysliak et al. [126]	BoHV-1 (Intranasal aerosol, 4 mL of 10^5^ TCID_50_/mL)	4 days	*M. bovis* (Intratracheal, 4 mL of 1.5 × 10^10^ CFU)	Coinfected group: higher weight loss and rectal temperature, higher rate of *M. bovis* isolation from blood of coinfected animals, more extensive lung lesions and shorter survival terms	4	Lack of BoHV-1 mono-infected group
Zhang et al. [130]	IDV (Intranasal, 10 mL of 10^7^ TCID_50_/mL)	5 days	*M. haemolytica* biotype A serotype 1 (Intratracheal, 30 mL of 10^9^ CFU)	Coinfected group: decreased clinical score compared to *Mannheimia* group, retarded viral shedding compared to IDV group; *Mannheimia* group: slightly increased lung lesions	2	
Lion et al. [131]	IDV (Nebulization, 10 mL of IDV 10^7^ TCID_50_/mL)	Simultaneous	*M. bovis* (Nebulization, 10^10^ CFU)	All infected groups: clinical signs present Coinfected group: earlier appearance and increased severity of clinical signs, gross lung lesions at 6 dpi	3	

*These studies were performed on a lamb model.

The first mixed infection studies were from 1960 to 1983, the majority being in vivo challenges using BPIV-3 or BoHV-1, the two viruses first associated with BRD, for the primary infection followed by inoculation with *M. haemolytica* [15, 92, 115–120]. In Jericho et al., two- to five-month-old calves exposed to aerosolized BoHV-1 then to *M. haemolytica* developed pneumonia when the delay between the viral and the bacterial infection was > 4 days. Calves infected solely with *M. haemolytica* did not develop severe pneumonia, underlining the importance of a viral pre-infection for the development of severe respiratory disease [118]. In Yates et al., six- to eight-month-old calves were exposed to BoHV-1 before being subsequently infected with *M. haemolytica* four to thirty days later. Although fibrinous pneumonia and pleuritis occurred in all four groups, animals exposed to the virus and bacteria four days apart had the most extensive and severe pathologic findings including foci of necrosis and/or focal areas of mucopurulent exudate on mucosal surfaces of the upper respiratory tract, with the pharyngeal tonsillar surfaces being most severely affected. Moreover, fibrinous pneumonia in coinfected calves resulted in the persistence of the viral antigen in the respiratory tract despite the resolution of the necrotic virus-induced lesions [119]. In contrast, a study by Carrière et al., did not observe any synergy in calves coinfected with the same pathogens, noting only mild lung lesions in all infected groups [120]. Similar findings were published by Saunders et al., where calves infected with BPIV-3 followed by different *Pasteurellaceae* species did not display increased respiratory disease severity, except increased nasal discharge [92].

Other experiments noted enhanced clinical signs when animals were pre-exposed to BVDV or BRSV before *M. haemolytica* or *H. somni* bacteria [121–126]. In Potgieter et al., two groups of six-month-old calves were inoculated at day 0 with either BVDV or *M. haemolytica* while a third coinfected group was inoculated first with BVDV and the subsequent bacterial pathogen 5 days later [121]. The authors reported pneumonic lesions reaching 2 to 15% of the total lung volume in the BVDV and *M. haemolytica* groups while the coinfected group developed severe fibrinopurulent bronchopneumonia and pleuritis comprising 40% to 75% of the total lung volume. In Gånheim et al., nine- to eighteen-month-old calves inoculated with either BVDV or *M. haemolytica* or coinfected with BVDV at day 0 and *M. haemolytica* 5 days later all had increased body temperature and depression, but the coinfected group had the most severe clinical signs with some animals not able to fully recover post-experimentation. The authors reported that both mono- and coinfected groups had similar magnitudes of acute phase proteins (AAPs) responses, particularly fibrinogen, haptoglobin and serum amyloid A, but the duration of elevated APPs expression was significantly longer in the BVDV/*M. haemolytica* group than in the BVDV group, reflecting the duration of clinical signs [122].

The first in vivo report of BRSV experimental infection in combination with *Pasteurellaceae* strains was actually performed in four-week-old lambs mono- or coinfected with BRSV or *M. haemolytica* at the same time. Pneumonic lesions were more frequent, extensive, and severe in coinfected lambs than in lambs inoculated with either agent alone. The authors postulated that BRSV compromised the lungs through the formation of lesions, promoting *M. haemolytica* establishment and subsequently, more severe pneumonic lesions than it could produce alone [127]. In the same animal model, similar findings were reported by Trigo et al. [124]. Later, in Gershwin et al., 9-month-old calves inoculated with a virulent strain of BRSV and *H. somni* 6 days later demonstrated significant mean clinical score differences compared to the groups infected with a single pathogen alone. Necropsy revealed severe bilateral consolidation in the anterior ventral lung lobes only in the coinfected group [128]. These results are in accordance to a similar coinfection study where calves pre-infected with BRSV and *H. somni* eight days later showed significantly more severe clinical signs and pneumonic lesions than animals inoculated with one pathogen alone [129].

In Prysliak et al., the pathogenicity of *M. bovis* was studied in six- to eight-month-old calves pre-exposed to BVDV or BoHV-1. Animals challenged with BoHV-1 prior to *M. bovis* inoculation 4 days later displayed weight loss, increased body temperature, and significantly shorter survival. At necropsy, the lungs of the BoHV-1/*M. bovis* group had extensive areas of bronchopneumonia, consolidation, and multifocal white nodules containing caseous material, whereas those from the *M. bovis* group displayed small consolidations without white nodules. No body weight loss was recorded for the BVDV/*M. bovis* group and there were no typical *M. bovis* pneumonia lesions found at necropsy [126].

As IDV was recently discovered to be a cattle pathogen, researchers started to investigate its possible role in BRD onset, assessing if IDV infection could worsen respiratory signs when co-inoculated with other pathogens in a manner similar to the viruses mentioned above. Four- to six-month-old calves infected with IDV at day 0 and *M. haemolytica* at day 5 had similar overall clinical scores as calves infected with IDV alone, while calves only infected with *M. haemolytica* had more severe gross lung lesions compared to the negative control group. *M. haemolytica* severe bronchopneumonia signs could not be reproduced in the coinfected calves suggesting that IDV and *M. haemolytica* coinfection does not alter the respiratory pathology of calves [130]. In another study, six-week-old calves were infected with either IDV, *M. bovis*, or IDV and *M. bovis* together [131]. Although the *M. bovis* group did not present bronchopneumonia and caseonecrotic lesions typical of *M. bovis* infection, the authors reported that the coinfected group had a shorter time span of presented clinical signs and significantly increased clinical score, as well as increased severity of trachea and lung macroscopic and microscopic lesions. Starting at 2 days post-infection, upregulated IFNγ levels were found in bronchoalveolar lavages from the coinfected group, reflecting increased leukocyte recruitment in the airway lumen. The authors also noted that *M. bovis* colonization of the lower respiratory tract was aided by the viral infection.

### In vitro approaches to further elucidate viral and bacterial coinfection pathogenicity mechanisms

Several studies attempt to explain the mechanisms underlying the enhanced pathology often observed during coinfection, mostly through in vitro approaches. One of the most well studied mechanisms of bacterial superinfection is the enhancement of bacterial adherence resulting from prior viral infection. In Sudaryatma et al., trachea, bronchus and lung primary cell lines were infected with BRSV before *P. multocida* [132]. The authors noticed that *P. multocida* adherence was greatly increased in pre-infected cells derived from the lower respiratory tract compared to cells that were not previously exposed to BRSV, together with an up-regulation of IL-6 mRNA expression. The same authors later reported an increased accumulation of the platelet-activating factor receptor (PAFR) in vitro and also demonstrated that *P. multocida* adherence depended on PAFR expression [133]. This work highlights a possible mechanism of bacterial superinfection caused by *P. multocida* following BRSV infection, that is often observed in field conditions [8]. In another recent work, the same authors observed an increase in *P. multocida* adherence following BCoV infection, noticing an increase in intercellular adhesion molecule-1 (ICAM-1) and PAFR, thus highlighting that the same mechanism could be shared among other BRD viruses [134]. In Agnes et al., infections with BRSV and superinfections with *H. somni* were carried out in BAT2 alveolar type 2 cell model [135]. The coinfection resulted in enhanced cytotoxicity for alveolar epithelial cells, increased transmigration of *H. somni* across the alveolar cell barrier, and matrix metalloproteinases MMP1 and MMP3 increased expression and activity. This could explain the observed results in their previous in vivo experiment, where they showed that *H. somni* and BRSV act synergistically in vivo to cause more severe bovine respiratory disease than either agent alone [128]. The same authors also reported, that BAT2 cell treatment with *H. somni* infected supernatants up-regulated antiviral genes and dramatically reduced a subsequent BRSV replication, showing once again that the timing of each pathogen infection is an important factor for the overall impact on pathology [136]. Finally, in McGill et al., the authors observed that in peripheral blood mononuclear cells (PBMC), coinfection with BRSV and *M. haemolytica* exacerbated IL-17 production, which plays a critical role in neutrophil recruitment and inflammation, a characteristic trait of *M. haemolytica* severe pasteurellosis in calves [137].

### Viral coinfections: a less explored model of increased pathogenesis in BRD

The “viral infection followed by bacterial superinfection” model seems to be the most frequent and best described dynamic in cattle herds. There is currently very little information about viral superinfections in BRD. After an exhaustive literature search, we found three in vivo studies investigating the impact of a primary viral infection followed by a second viral infection [138–140]. BVDV was used in the three studies as the primary viral infection, likely due to its immunosuppressive nature [141]. We also identified two other studies investigating the impact of simultaneous BRSV and BVDV coinfection [142, 143]. All in vivo viral/viral respiratory coinfection calf studies are summarized in Table 2.

**Table 2:** **In vivo studies from the scientific literature performed on young calves to assess viral coinfections impact on BRD**

Reference	Primary viral challenge (route of infection and dose/animal)	Time between exposure to the two pathogens	Secondary viral challenge (route of infection and dose/animal)	Main results of the clinical trials	Impact of the coinfection on BRD (score 1 to 4)	Study limitations
Pollreisz et al. [142]	BVDV-1 (Intranasal and intratracheal, 5 mL of 2 × 10^8^ TCID_50_)	Simultaneous, 1 day and 2 days	BRSV (Intranasal and intratracheal, 5 mL of 10^6^ TCID_50_/mL)	BVDV group: mild signs BRSV group: serous nasal discharge, rapid and shallow respiration and depression Coinfected group: excessive serous or mucopurulent nasal discharge, rapid breathing, diarrhoea, severe depression, one calf had to be euthanized	4	
Brodersen et al. [143]	BVDV	Simultaneous	BRSV	Coinfected group: increased clinical signs, higher viral shedding and increased lung lesions than infection with either virus alone	3	
Elvander et al. [138]	BVDV (non-cytopathogenic) (Intratracheal, 10^5^ TCID_50_/mL)	Simultaneous	BRSV (Intratracheal, BRSV group: 10 mL of 10^4^ TCID_50_/mL, coinfected group: 10 mL of BRSV 10^5^ TCID_50_/mL)	No increase in clinical signs in coinfected group	2	Lack of BVDV group; different BRSV dose in mono-infected and coinfected groups
Risalde et al. [145]	BVDV-1 (non-cytopathogenic) (Intranasal, 1 mL/nostril of 10^5^ TCID_50_/mL)	12 days	BoHV-1.1 (Intranasal,1 mL/nostril of BoHV-1.1 × 10^7^ TCID_50_/mL)	Appearance of clinical signs in all groups but increase in severity in coinfected group; increase in pro-inflammatory cytokines and APPs in coinfected group (IL-1β) and more severe inflammatory lesions	3	Lack of BVDV group
Ridpath et al. [140]	BVDV-2a (Intranasal aerosol, 4 mL of 10^6^ TCID_50_/mL)	3, 6 and 9 days	BCoV (Intranasal aerosol)	BCoV group: pyrexia but no gross lesions Coinfected group: higher fever, lung lesions present in all infected groups but more pronounced in 6-day delay group; peripheral blood lymphocytes count returned to baseline in 6-day delay group but not in 9-day delay group	3	BCoV dose is not reported
Ridpath et al. [140]	BCoV (Intranasal aerosol)	3 days	BVDV-2a (Intranasal aerosol, 4 mL of 10^6^ TCID_50_/mL)	BCoV group: pyrexia but no gross lesions Coinfected group: pyrexia and lung lesions in some coinfected calves consisting in pale, firm foci randomly scattered throughout the lungs but particularly obvious in the ventral caudal lobes	2	BCoV dose is not reported

In Pollreisz et al., nine- to twelve-month-old calves simultaneously infected with BRSV and BVDV developed more severe clinical signs, including fever and diarrhoea, and lung lesions than their mono-infected counterparts. In addition, coinfected calves had a longer duration of viral shedding in nasal secretions and higher infectious titres compared to the groups infected with BRSV or BVDV alone [142]. An in vitro study performed on alveolar macrophages demonstrated that concomitant infection with BRSV and BVDV suppressed alveolar macrophage functionality [144], potentially explaining the increased lung lesions observed in Pollreisz et al. [142]. In contrast, Elvander et al. reported no change in clinical signs in three-month-old calves concurrently infected with BVDV and BRSV [138].

In Risalde et al., eight-month-old calves pre-inoculated with a non-cytopathic BVDV strain followed by BoHV-1 inoculation twelve days later had more intense clinical signs and lesions, correlating with greater TNFα secretion and reduced IL-10 production than animals inoculated with BoHV-1 alone. Delayed IFNγ production and low IL-12 levels were also observed in coinfected animals [145]. In a following paper, the same authors described important lung vascular alterations produced by fibrin microthrombi and platelet aggregations within the blood vessels that were earlier and more severe in the BVDV and BoHV-1 coinfected group, suggesting that coinfection facilitates a procoagulant environment modulated by inflammatory mediators such as significantly decreased iNOS expression released by pulmonary macrophages [146]. In two subsequent studies, the same authors reported that coinfected animals displayed inhibited CD8 + and CD4 + T lymphocyte responses against BoHV-1, suggesting that BVDV pre-infection could impair local cell-mediated immunity to secondary respiratory pathogens [139] and provoke thymic lesions that temporarily downregulate Foxp3 lymphocytes and TGFβ expression and medullary CD8 + T cells development [147].

In Ridpath et al., BVDV and BCoV dual infection studies in vivo were performed using different sequences and delays of superinfection to assess pathogenicity. Calves inoculated with BVDV followed by BCoV 6 days later displayed more pronounced clinical signs and lung lesions compared to 3 days of delay, demonstrating that the timing of the secondary infection along with the pathogen itself plays an important role in coinfection pathogenesis [140]. In the same study, calves were also inoculated with BCoV followed by BVDV 3 days later but clinical signs and lung lesions were not as pronounced as in animals pre-infected with BVDV followed by BCoV challenge, questioning the role of BCoV as BRD initiator.

The in vivo studies described above support the notion that BVDV pre-infection aggravates the respiratory pathology induced by other viruses in a manner similar to bacterial superinfections, as previously discussed above. What the field lacks is data concerning other viral coinfections involved in BRD. For example, BRSV, also known to modulate host immune responses [148], could play a similar role, despite the absence of experimental in vivo evidence during respiratory coinfections. The small number of available studies on viral superinfections limits our understanding of the role of viruses in priming the immune system before causing a subsequent viral superinfection.

### Bacterial coinfections: can bacteria initiate BRD without the presence of primary triggers?

Contrary to viral/bacterial coinfections, bacterial coinfection models have been rarely explored in BRD studies. Multiple bacterial respiratory pathogens are often simultaneously detected from sick animals [8]. Despite this, respiratory bacteria interactions remain unclear. Some are part of the normal microbial flora of the upper respiratory tract of healthy animals (notably *Pasteurellaceae* family members) but are also often isolated from animals with respiratory signs [8, 49]. Different experimental in vivo infections with single bacterial challenge have been carried out throughout the years in calf models [35, 52, 149]. However, reproducing classical bronchopneumonia signs has been highly variable. Inoculation of the A3 serotype of *P. multocida* in calves induced clinical signs and lung lesions [35, 150, 151] whereas buffalo are susceptible to the A1 serotype [37]. In contrast, other studies reported milder lesions and overall pathology [152]. Animals experimentally infected with *M. haemolytica* alone either fail to develop bronchopneumonia [118, 120, 121, 130], or manifest severe clinical illness and reach end-point limits during the study [149, 153]. Such confounding study differences could be due to intrinsic characteristics of the animals (immune status, age and breed) as well as differences in the bacterial strains that are not yet known and therefore, could not be accounted for the highly controlled experiments.

We retrieved two in vivo studies from the literature investigating the impact of dual bacterial infection in calves. In Houghton and Gorlay, calves simultaneously inoculated with *M. bovis* and *M. haemolytica* were more severely affected than animals inoculated with only one pathogen. Vast differences were seen during necropsy with coinfected animals displaying 34 to 55% of lung consolidation compared to only 1–6% for calves from the *M. bovis* group and 0–1% for calves from the *M. haemolytica* group [154]. Subsequently, the same authors performed different dual bacterial challenges in gnotobiotic calves [155]. Calves were first inoculated with *M. bovis* followed by *M. haemolytica* one or two days later. Two animals also received a *M. haemolytica* strain that was cultured for 18 h previously to the challenge and two others received a strain that was cultured for 6 h. No clinical signs were reported for the calf infected with only *M. haemolytica*, whereas calves that were inoculated with *M. haemolytica* 2 days later displayed severe illness and 16% of lung consolidation at necropsy. However, calves that received the second pathogen one day later were more ill compared to the group inoculated 2 days later. In addition, high lung consolidation (50–64%) was reported for this group. Two calves inoculated with *M. haemolytica* then *M. bovis* two days later only developed mild signs without pneumonia. Similar challenges were performed on conventionally reared calves, with simultaneous inoculation of *M. bovis* and *M. haemolytica*, or, inoculation by *M. bovis* first followed by *M. haemolytica* one day later. Calves in the *M. haemolytica* group did not display any lesions or illness and only a few animals in the coinfected group had fever and 6–8% of lung consolidation at necropsy. In contrast, calves first dosed with *M. bovis* followed by *M. haemolytica* one day later had severe respiratory signs, resulting in the death of one calf and high lung consolidation (28 to 60%). The *M. bovis* group showed moderate clinical signs and less lung consolidation (27–40%). These data underline the relationship between coinfection and the development of severe pneumonia [155]. This is in agreement with another study, where the death of two gnotobiotic calves was reported 24 h followed simultaneous inoculation with *M. bovis* and *M. haemolytica* [156]. Table 3 summarizes the calf in vivo studies on bacterial respiratory coinfections.

**Table 3:** **In vivo studies from the scientific literature performed on young calves to study bacterial coinfections impact on BRD**

Reference	Primary bacterial challenge (route of infection and dose/animal)	Time between exposure to the two pathogens	Secondary bacterial challenge (route of infection and dose/animal)	Main results of the clinical trials	Impact of the coinfection on BRD (score 1 to 4)	Study limitations
Houghton and Gorlay, [154]	*M. bovis* (Intranasal)	Simultaneous	*M. haemolytica* (Intranasal)	Dually inoculated animals were more severely affected than animals inoculated with one pathogen *M. bovis* group: 1–6% lung consolidation *M. haemolytica* group: 0–1% lung consolidation Coinfected animals displayed 34 to 55% of lung consolidation	3	
Gourlay et al. [155] (gnotobiotic calves)	*M. bovis* (Intranasal and intratracheal, 6 × 10^8^ CFU)	1 day and 2 days	*M. haemolytica* (Intranasal and intratracheal, 7 × 10^8^ CFU cultured for 6 h or for 18 h before inoculation)	*Mannheimia* group: no illness or gross lesions Coinfected group: severe clinical illness and 16% of lung consolidation at necropsy with 2-days delay, severe illness and high area of lung consolidation in dually infected calves (50–64%) inoculated with 1-day delay between pathogens, increased lesions when using 6 instead of 18 h culture of *Mannheimia*	3	Lack of *M. bovis* group
Gourlay et al. [155] (gnotobiotic calves)	*M. haemolytica* (Intranasal and intratracheal, 7 × 10^8^ CFU cultured for 6 h or for 18 h before inoculation)	2 days	*M. bovis* (Intranasal and intratracheal, 6 × 10^8^ CFU)	No significant increase in pneumonia in coinfected animals, mild signs in all groups	2	Lack of *M. bovis* group
Gourlay et al. [155] (Conventionally reared calves)	*M. bovis* (Intranasal and intratracheal, 6 × 10^8^ CFU)	Simultaneous	*M. haemolytica* (Intranasal and intratracheal, 7 × 10^8^ CFU cultured for 6 h or for 18 h before inoculation)	*Mannheimia* group: no illness and no gross lung lesions Coinfected group: only few calves displayed fever and clinical signs and 6–8% of lung consolidation at necropsy, the remaining calves displayed no clinical signs	2.5	
Gourlay et al. [155] (Conventionally reared calves)	*M. bovis* (Intranasal and intratracheal, 6 × 10^8^ CFU)	1 day	*M. haemolytica* (Intranasal and intratracheal, 7 × 10^8^ CFU cultured for 6 h or for 18 h before inoculation)	*M. bovis* group: moderate clinical signs and reduced extent of lung consolidation (27–40%) Coinfected group: severe clinical signs resulting in the death of one calf and higher extent of lung consolidation (28 to 60%)	4	

### Bacterial coinfection studies: synergy or antagonism?

Currently, few in vitro studies investigating the interactions among different bacteria exist. In Corbeil et al., different bacterial strains (*P. multocida*, *M. haemolytica* and *H. somni*) isolated from bovine microbial flora were grown together to examine whether they would inhibit or enhance their growth [157]. The authors discovered that the majority of microbial strains could enhance the growth of the tested pathogens, especially those from the *Micrococcus*, *Corynebacterium* and *Staphylococcus* genera, whereas a discrete number of isolates did not affect their growth. In contrast, only some *Bacillus* genus strains could inhibit *Pasteurellaceae* growth. In Bavananthasivam et al., the authors tested growth competition between *P. multocida* and *M. haemolytica* and found that each showed similar growth when cultured together but upon physical separation by a membrane, *M. haemolytica* growth was inhibited by a contact-proximity mechanism [158], similar to what was already observed for *Bibersteinia trehalosi* in sheep pneumonia [159], hypothesizing that the inhibition occurred though similar molecular mechanisms. Inhibition of *M. haemolytica* by probiotic bacteria was also demonstrated in vitro [160]. Since previous studies reported that *P. multocida* can be isolated from the lower respiratory tract from calves experimentally infected with *H. somni* [161] but also during natural cases of BRD [49], the co-existence of *H. somni* and *P. multocida* in polymicrobial film was investigated in vitro and in vivo [162]. In the in vitro model, both pathogens were shown to co-exist and to contribute to biofilm formation. Two eight-week-old calves were then intratracheally challenged with 10^9^ CFU of *H. somni* so that lung tissues could be analysed for polymicrobial formation. Both pathogens were detected by PCR in the lungs, supporting the hypothesis that *H. somni* and *P. multocida* can cohabit in polymicrobial films in vivo. In another study, the carriage of *H. somni*, *P. multocida* and *M. haemolytica* was assessed by qPCR from nasal swabs collected from healthy beef calves (*n* = 60) during a 75-day study [163]. Co-carriage of two or three bacterial species was detected in 47 animals but *P. multocida* remained the most prevalent during the entire study, either as co-carriage with *H. somni* occurring the most frequently followed by *M. haemolytica* and lastly with *H. somni*. Taking all the experimental evidence into consideration, we cannot conclude whether a synergistic or antagonistic effect is present among different *Pasteurellae* bacterial strains*.* Further studies are needed to investigate the interactions among these pathogens in the context of BRD.

## Discussion

In this review, we consolidated experimental evidence describing coinfection mechanisms potentiating pneumonia aetiology in cattle. The most studied mechanism of BRD onset in calves is the primary viral infection followed by a secondary bacterial superinfection model, with evidence suggesting it to be one of the most common scenarios triggering BRD. Several in vivo experiments showed that a primary viral infection impacts *M. haemolytica* superinfection. The viruses that seem to enhance secondary bacterial infection the most include BRSV, BVDV and BoHV-1 with mean scores higher than 3. BPIV-3 received a mean score of 2.67, also indicating a close association. Despite this, no solid conclusions can be drawn due to the very limited number of undertaken studies. In addition, two of the BRSV studies were performed in lambs, not in calves. A few studies using *P. multocida*, *H. somni* and *M. bovis* as bacterial secondary infection could be retrieved, with the highest impact score for BoHV-1 followed by *M. bovis* (mean score of 4) and BRSV followed by *H. somni* (mean score of 3.5). Multiple in vitro studies showed that viral priming increased bacterial adherence and colonization of the respiratory tract, suggesting a possible mechanism underlying the onset of bronchopneumonia in cattle. This could explain why viruses and bacteria are often co-detected in the respiratory tract of field animals with BRD signs. A limited number of viral coinfection studies (*n* = 4) was also retrieved, showing that a primary viral infection increases the pathogenicity of a secondary viral infection. Despite this, only the role of BVDV has been explored throughout the years for viral coinfections. The mechanisms utilized by other viral pathogens such as BCoV and IDV remain unclear. One of the most important questions concerning the dynamics of bacterial derived respiratory infection is whether contagious spread between animals stems from bacterial replication in the lungs or whether said bacteria is already present in the nasopharynx, accessing the lower respiratory tract when immune responses are impaired from a primary trigger (the secondary bacterial superinfection model).

A few studies have attempted to adress this question. Young bulls (*n* = 112) arriving at a fattening facility were divided into different pens and observed for 40 days. Nasal swabs and transtracheal aspirations were collected to detect *M. haemolytica* and to study the clonal diversity between the upper and lower respiratory tracts. During the BRD outbreaks that occurred at the facility, *M. haemolytica* was frequently isolated from sick animals with 75 bulls testing positive during the study. Among these, *M. haemolytica* was cultured from transtracheal aspirates from 23 asymptomatic bulls. Pulse field gel electrophoresis (PFGE) analysis revealed a moderate agreement in clone diversity within nasal swabs and transtracheal aspirates within the same animals but high within-pen diversity, indicating that the disease was due to predisposing triggers enabling the bacteria to overcome the animal immune system and the normal flora. Despite this, the authors observed horizontal gene transfers from bulls in the nearest pen as well [24]. High genetic diversity within the same feedlot was also observed in other studies for *M. haemolytica* [164] and for *P. multocida* [165]. These results suggest that BRD episodes associated with these pathogens are probably due to predisposing factors overcoming the normal flora than the spread of a contagious clone among animals within a herd. Young pre-weaned calves recently arrived to feeder farms are exposed to high stress levels, likely the most important trigger to BRD aetiology.

A separate evaluation should be made for *M. bovis*, as this pathogen is not part of the commensal flora of healthy animals. In experimental conditions, a primary *M. bovis* challenge followed by *M. haemolytica* one day later increased the severity of illness compared to calves singly challenged or simultaneously challenged with both pathogens [155]. The conditions of the experimental infection do not represent the reality of animals in the field within a herd (the pathogens are challenged intratracheally with a high infectious dose), however these data suggest that *M. bovis* could potentially initiate BRD development.

Different in vitro studies tried to elucidate bacterial pathogenic interactions; however, mechanisms of synergy or antagonism among the studied bacterial strains remain unknown as there are too few studies, leaving a gap in knowledge about the polymicrobial aetiology of BRD.

In this study, we developed a scoring system to evaluate the impact of coinfection on overall cattle BRD pathology. This scoring system is meant to generalize the effects of specific pathogen pairs during coinfection with the caveat that there are major limits obfuscating the true impact, including poorly described control groups in certain studies and differences in induced respiratory pathology upon challenge of the same pathogen among all the studies. For example, inoculation with *M. haemolytica* induced BRD in some studies but not others, making it difficult to compare the true impact of *M. haemolytica* during coinfection. High heterogeneity across studies leads to additional difficulties when comparing results as parameters considerably change from one study to another, notably the infection route or pathogen dose, the assays used to confirm infection and seroconversion, and the age and breed of the animals. In addition, in vivo studies assessing the impact of coinfections among respiratory pathogens in cattle are limited, as are the number of animals used per study. One way to control for error is by using specific-pathogen free (SPF) calves, negating confounding effects associated with animals previously exposed to different pathogens and immunologically primed to combat infection, potentially resulting in altered pathological changes upon challenge.

Few studies (*n* = 7) have attempted to study coinfections using alternative models to animal testing. The onset of new in vitro, ex vivo or in-vivo-like models in recent years could represent a valid replacement for primary studies before confirmation in animals. In particular, primary cell cultures, tissue cultures, organ slices and organoids provide a good start to change, both addressing the 3 R’s principle (Reduction, Replacement and Refinement) and expanding the global scientific field (Figure 1).

Over the course of the 20^th^ and the twenty-first century, the impact of different pathogens on BRD has changed. On one side, the development of prophylactic measures has helped control some infectious diseases in cattle, as notably shown by the eradication program for IBR and BVDV [86]. On the other hand, new emerging pathogens continue to appear, probably due to intensified cattle farming from the twentieth century like the appearance of high-density animal feedlots. New pathogens potentially involved in BRD that were not considered before (i.e. Influenza D virus) can be quickly discovered through NGS [166], potentially leading the way for an early risk assessment surveillance program in which cattle herds are monitored for emerging pathogens in order to prevent their circulation. New techniques like NGS facilitate studies on respiratory pathogenic interactions with the surrounding normal bacterial species as well as the mechanisms underlying the pathogenesis of respiratory disease in cattle. During surveillance, longitudinal studies could also be conducted to observe the dynamics of respiratory outbreaks caused by mixed infections, providing insight about the timing of pathogen introduction during BRD development (Figure 2).

## Supplementary Information

**Additional file 1.** **Description of the scoring system criteria to evaluate the impact of coinfections on BRD.**

## Data Availability

Data sharing not applicable to this article as no datasets were generated or analysed during the current study.
