# Supplementary material for: Understanding the mechanisms of viral and bacterial coinfections in bovine respiratory disease: a comprehensive literature review of experimental evidence
Source: Vet Res. 2022 Sep 6;53:70. doi: 10.1186/s13567-022-01086-1 (PMC9449274; doi:10.1186/s13567-022-01086-1)
Supplement: Supplementary file 1 — Additional file 1. Description of the scoring system criteria to evaluate the impact of coinfections on BRD. [file 13567_2022_1086_MOESM1_ESM.docx]

**Additional file 1 Scoring system to evaluate the impact of co-infections on BRD**

In this review, we developed a scoring system (ranging from 1 to 4) to describe the impact of co-infections on the overall animal pathology applied to all in vivo studies retrieved from the scientific literature based on the following criteria:

1= infection did not result in any noticeable clinical signs in either single or dually infected animal models.

2= mono-infection resulted in at least one of the following clinical signs: fever (≥ 39.9 °C), increased nasal discharge (serous or mucopurulent), cough, tachypnea, abnormal lung sounds upon auscultation, depression, weight loss, loss of appetite, and gross lung lesions. Clinical signs or lung lesions were present in single challenged groups, but co-infection did not increase the severity of the pathology.

3= mono-infection resulted in multiple clinical signs including: fever (≥ 39.9 °C), increased nasal discharge (serous or mucopurulent), cough, tachypnea, abnormal lung sounds upon auscultation, depression, weight loss, loss of appetite, and gross lung lesions. Clinical signs and gross lung lesions persisted longer and were more severe in the co-infected group.

4= mono-infection resulted in multiple clinical signs including: fever (≥ 39.9 °C), increased nasal discharge (serous or mucopurulent), cough, tachypnea, abnormal lung sounds upon auscultation, depression, weight loss, loss of appetite, and gross lung lesions. A minimum of one co-infected animal had to be euthanized or succumbed to the disease during or at the end of the study.
